# Enabling Multi-Chemisorption Sites on Carbon Nanofibers Cathodes by an In-situ Exfoliation Strategy for High-Performance Zn–Ion Hybrid Capacitors

**DOI:** 10.1007/s40820-022-00839-z

**Published:** 2022-04-15

**Authors:** Hongcheng He, Jichun Lian, Changmiao Chen, Qiaotian Xiong, Cheng Chao Li, Ming Zhang

**Affiliations:** 1 Key Laboratory for Micro/Nano Optoelectronic Devices of Ministry of Education, Hunan Provincial Key Laboratory of Low-Dimensional Structural Physics and Devices, Hunan Joint International Laboratory of Advanced Materials and Technology for Clean Energy, School of Physics and Electronics, College of Semiconductors (College of Integrated Circuits), Hunan University, Changsha, 410082 People’s Republic of China; 2 Tsinghua Shenzhen International Graduate School, Tsinghua University, Shenzhen, 518055 People’s Republic of China; 3 School of Chemical Engineering and Light Industry, Guangdong University of Technology, Guangzhou, 510006 People’s Republic of China

**Keywords:** Nitrogen doping, Carbonyl functionalization, Chemisorption sites, Flexible, Zn-ion hybrid capacitors

## Abstract

Carbon nanofibers films are typical flexible electrode in the field of energy storage, but their application in Zinc-ion hybrid capacitors (ZIHCs) is limited by the low energy density due to the lack of active adsorption sites. In this work, an in-situ exfoliation strategy is reported to modulate the chemisorption sites of carbon nanofibers by high pyridine/pyrrole nitrogen doping and carbonyl functionalization. The experimental results and theoretical calculations indicate that the highly electronegative pyridine/pyrrole nitrogen dopants can not only greatly reduce the binding energy between carbonyl group and Zn^2+^ by inducing charge delocalization of the carbonyl group, but also promote the adsorption of Zn^2+^ by bonding with the carbonyl group to form N–Zn–O bond. Benefit from the multiple highly active chemisorption sites generated by the synergy between carbonyl groups and pyridine/pyrrole nitrogen atoms, the resulting carbon nanofibers film cathode displays a high energy density, an ultralong-term lifespan, and excellent capacity reservation under commercial mass loading (14.45 mg cm^‒2^). Particularly, the cathodes can also operate stably in flexible or quasi-solid devices, indicating its application potential in flexible electronic products. This work established a universal method to solve the bottleneck problem of insufficient active adsorption sites of carbon-based ZIHCs.Imoproved should be changed into Improved.

**Supplementary Information:**

The online version contains supplementary material available at 10.1007/s40820-022-00839-z.

## Highlights

- The high pyridine/pyrrole nitrogen-doped and carbonyl-functionalized nanosheets on flexible electrospun porous carbon nanofibers film cathode integrates the advantages of large specific surface area, strong hydrophilicity and high Zn^2+^ adsorption activity.
- The Zinc-ion hybrid capacitors exhibits a high capacitance retention of 99.2% over 200,000 cycles at 40 A g^−1^.
- A synergistic Zn^2+^ storage mechanism between carbonyl and pyridine/pyrrole nitrogen atoms is proposed.

**Supplementary Information:**

The online version contains supplementary material available at 10.1007/s40820-022-00839-z.

## Graphical abstract

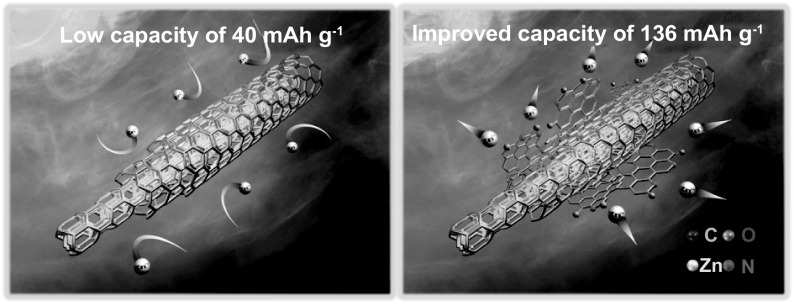

## Introduction

With the growing problem of carbon dioxide emissions from excessive consumption of fossil fuels, there is an urgent need to develop a sustainable clean energy storage system to achieve carbon–neutral development strategies [1, 2]. Among various energy storage systems, the aqueous zinc ion hybrid capacitors (ZIHCs), which is usually composed of porous carbon cathode and zinc metal anode, has recently attracted a great deal of research due to its advantages of environment-friendly, high safety, low cost and long-term durability [3–5]. However, the relatively low energy density still hinders its practical application. Considering that zinc metal has a theoretical capacity of 820 mAh g^−1^, it is able to generate sufficient Faraday reaction during the process of charging and discharging to match the maximum capacitance of the porous carbon cathode [6, 7]. Therefore, improving the capacitance of carbon cathode is the key factor to increase the energy density of ZIHCs [8].

The capacitance of the carbon cathode consists of two parts: electric double-layer capacitance (EDLC) and pseudocapacitance (Faradaic redox reactions between the electroactive substance on the carbon surface and the electrolyte) [9, 10]. So far, a variety of carbon cathodes have been developed around these two perspectives to increase the energy density of the ZIHCs, such as activated carbon with large specific surface area [11, 12], porous carbon with rich microporous structure [13, 14], and heteroatom-modified carbon with pseudocapacitive active [15–17]. In practical applications, those treated carbon cathodes need to be coated on a current collector, such as carbon cloth or carbon paper, to achieve effective energy transmission [18]. However, the carbon fiber-based current collector itself has poor electrochemical performance due to its low specific surface and lack of active adsorption sites on the surface [16, 19]. The use of such a current collector with low electrochemical activity is not conducive to increasing the overall energy density of the ZIHCs [20]. In addition, the shedding of electroactive materials from the current collector is also a key factor in the performance degradation of ZIHCs [21, 22]. Thus, the manufacturing process of the electrode will be simplified greatly and the use of electrochemically inactive binders also will be avoided if abundant active adsorption sites can be in-situ constructed on the surface of the carbon fiber current collector which are directly used as a flexible cathode of ZIHCs. At the same time, due to its in-situ nature, the stability of the carbon cathode will be improved.

**Scheme 1:**
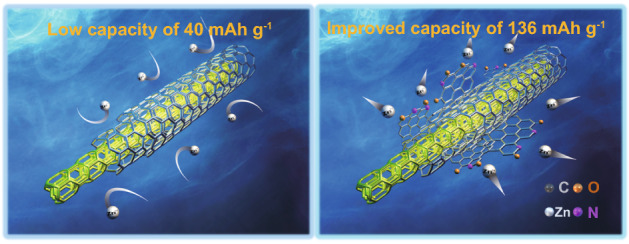
In situ exfoliation of the high pyridine/pyrrole N-doped and carbonyl-functionalized nanosheets on the carbon nanofiber

In this work, an in-situ exfoliation strategy was proposed to introduce the high pyridine/pyrrole nitrogen-doped and carbonyl-functionalized nanosheets on flexible electrospun porous carbon nanofibers film. The resulting porous carbon nanofibers film (labeled as N-OPCNF) with multi-chemisorption sites was used as a free-standing cathode for ZIHCs. As shown in Scheme 1, the carbon nanofibers film cathode with a complete structure only exhibited a capacity of 40 mAh g^−1^ at 0.1 A g^−1^ because there are few active sites to adsorb Zn^2+^. After exfoliating the high pyridine/pyrrole nitrogen doped and carbonyl functionalized nanosheets, the complete structure of the carbon nanofibers is partially opened, so that Zn^2+^ can accumulate and diffuse on the whole carbon nanofibers. The specific capacity of the N-OPCNF electrode obtained by this strategy is increased to 136 mAh g^−1^, which is 3.4 times that of the original electrode. In addition, N-OPCNF cathode exhibits a high capacitance retention of 99.2% over 200,000 cycles at 40 A g^−1^. The experimental results and theoretical calculations prove that the enhanced electrochemical activity originates from the multiple highly active chemisorption sites generated by the synergistic effect between the pyridine/pyrrole N atoms and carbonyl groups on the exfoliated surface.

## Experimental Section

### Preparation of N-OPCNF

Firstly, 0.87 g zinc acetate dihydrate (Aladdin) and 0.24 g melamine (Aladdin) were added to 6 mL N, N-dimethylformamide (Aladdin) and stirred for 0.5 h. Then, 0.43 g polyacrylonitrile (Sigma-Aldrich) were added and stirred for 12 h. The mixture solution was injected into a plastic syringe (10 mL) and electrospun with a flow rate of 0.6 mL h^−1^, a high voltage of 15 kV and a distance between the spinneret and flat receiver of 18 cm. The collected electrospun fibers films were heated to 230 °C for 3 h in a muffle furnace. Thereafter, the nitrogen-doped porous carbon nanofibers (N-PCNF) film was prepared via carbonization the pre-oxidized products at 950 °C for 2 h under H_2_ (10%)/Ar atmosphere. After that, the N-PCNF film was immersed in 30 mL concentrated nitric acid and maintained at 90 °C for 5 h. Finally, the nitrogen-doped and carbonyl-functionalized porous carbon nanofibers (N-OPCNF) film was obtained by washed the N-PCNF film treated with nitric acid several times with deionized water. As a comparison, carbonyl-functionalized porous carbon nanofibers (OPCNF) were prepared without the addition of melamine following the same treatment conditions. Carbonyl-functionalized carbon nanofibers (OCNF) were prepared without the addition of melamine and zinc acetate dihydrate following the same treatment conditions.

### Electrochemical Measurements

The working electrode was made directly by the flexible and binder-free N-OPCNF film. The mass density of the working electrodes was about 0.8–1.5 mg cm^−2^. Electrodes with mass loads from 4.45 to 14.45 mg cm^−2^ can be obtained by adjusting the thickness of the N-OPCNF film. All the as-prepared films were assembled into CR2032-type cells under air atmosphere. Zn mental was used as the anode and 1 m ZnSO_4_ aqueous solution was used as the electrolyte. A Neware battery testing system was used to test the galvanostatic charge/discharge process within a voltage range of 0.2–1.8 V. Cyclic voltammetry and electrochemical impedance spectroscopy were carried out on a CHI 660E workstation.

### Assembly of Quasi-Solid-State ZIHCs

The gel electrolyte is prepared by adding 0.2 g of gelatin to 1 m ZnSO_4_ aqueous solution (10 mL), followed by continuous stirring at 80 °C for 5 h [23, 24]. Then, gelatin/ZnSO_4_ gel electrolyte and a Whatman glass fibers separator were sandwiched between N-OPCNF cathode and Zn anode to fabricate the quasi-solid ZIHC device. The entire device was encapsulated with polyimide tape.

### Computational Details

First-principles calculations of all structural models considered in this work were based on density functional theory (DFT) as implemented in the Vienna Ab-initio Simulation Package (VASP). The exchange–correlation interaction between electrons was described by the Perdew-Burke-Ernzerhof (PBE) functional under generalized gradient approximation (GGA). Projector augmented wave (PAW) method with a plane-wave cutoff energy of 500 eV was used to wave functions expansion. Electrons in orbits of 3*d*4*s* for Zn, 2*s*2*p* for C, N, O, and 1*s* for H were deemed as valence electrons according to the pseudopotential method. Gaussian smearing method with a width of 0.05 eV and Monkhorst–Pack scheme with a k-point density of about 2π × 0.03 Å^−1^ were employed for Brillouin zone integrations. The charge density was optimized until the total energy was less than 10^–5^ eV, and the atomic positions were relaxed to all forces were no greater than 0.02 eV Å^−1^. The charge transfers between atoms were calculated using a Bader analysis program. To weaken the artificial Coulomb interaction between different cells caused by the enforcement of periodic boundary conditions in a supercell approach, vacuum layers with a thickness about 12 Å were added along aperiodic axises.

## Results and Discussion

### Synthesis and Structure Characterizations

**Fig. 1:**
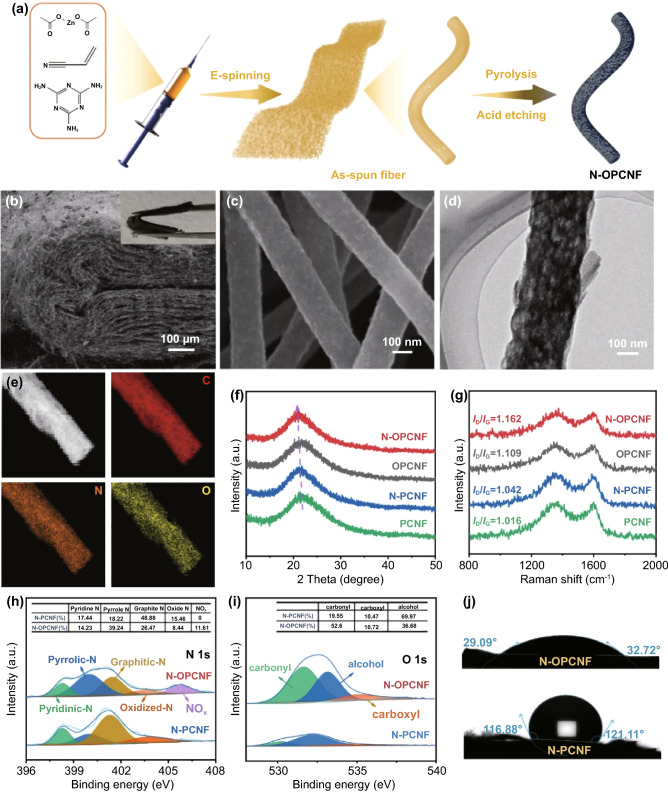
**a** Schematic illustration, **b**, **c** SEM image, **d** TEM image, **e** high-angle annular dark-field STEM image and corresponding elemental mapping of N-OPCNF. **f** XRD pattern and **g** Raman spectra of N-OPCNF, OPCNF, N-PCNF, and PCNF. **h** High-resolution N 1*s* and **i** O 1*s* XPS spectra of N-OPCNF and N-PCNF. **j** Contact angles of a water droplet on N-OPCNF and N-PCNF samples

The preparation process of the N-OPCNF is illustrated in Fig. 1a. Firstly, the polymer fibers film containing zinc species and melamine was obtained by electrospinning. Following sequential pre-oxidation, carbonization, the melamine and PAN in the polymer fibers film would decompose to form a flexible N-doped carbon nanofibers structure. At the same time, the zinc species will evaporate under high temperature conditions, resulting in a rich pore structure in the N-doped carbon nanofibers, and the resulting sample labeled as N-PCNF. Finally, the N-PCNF film was oxidized with concentrated nitric acid, and then the high pyridine/pyrrole N-doped and carbonyl-functionalized nanosheets was exfoliated on its surface (N-OPCNF). For comparison, to study the importance of the nitrogen additives, porous carbon nanofibers without melamine as an additional nitrogen source were synthesized and labeled as PCNF. PCNF that has been oxidized with nitric acid is labeled OPCNF. Scanning electron microscope (SEM) images show that all samples are composed of one-dimensionally interconnected carbon nanofibers (Figs. 1c and S1a–c), and the N-OPCNF film exhibits excellent flexibility at a bending angle of 180° (Fig. 1b). Compared with PCNF (Fig. S1a) and N-PCNF (Fig. S1b), the nitric acid etching made the surfaces of OPCNF (Fig. S1c) and N-OPCNF (Fig. 1c) rougher, indicating that the surfaces of the two samples have formed abundant edge structures. In addition, cross-sectional SEM images (Fig. S2a-b) showed that the core of carbon nanofibers remained intact after nitric acid etching. Transmission electron microscope images clearly show the surfaces of OPCNF (Fig. S1f) and N-OPCNF (Fig. 1d) have exfoliated graphene-like carbon nanosheets. In contrast, PCNF and N-PCNF that were not etched with nitric acid showed a smooth surface structure (Fig. S1d-e). The element mapping of N-OPCNF (Fig. 1e) indicate that C, O, and N are uniformly distributed on the surface of the carbon nanofibers, which proves the successful introduction of oxygen and nitrogen in the carbon nanofibers.

X-ray diffraction (XRD) measurements of the four samples (Fig. 1f) showed a broad peak corresponding to the graphitic carbon (002) plane. The (002) peaks of N-OPCNF, OPCNF, N-PCNF, and PCNF are located around 20.91°, 21.67°, 21.31°, and 21.83°, respectively, corresponding to the layer spacing of 0.425, 0.41, 0.416, and 0.407 nm. The expanded interlayer spacing of N-OPCNF can be attributed to the doping of N atoms and the introduction of carbonyl groups in the carbon nanofibers [25, 26]. The peak intensity ratio (*I*_D_/*I*_G_) of *sp*^3^-type disordered carbon (D band) and *sp*^2^-type graphitized carbon (G band) of Raman spectroscopy was used to study the structural disorder and defects of carbon nanofiber films (Fig. 1g). The *I*_D_/*I*_G_ of N-OPCNF (1.162) is higher than OPCNF (1.109), N-PCNF (1.042), and PCNF (1.016), indicating that in-situ exfoliation of N-doped and carbonyl-functionalized nanosheets give N-OPCNF more structural defects [27, 28]. In addition, the stripped carbon nanosheets also give N-OPCNF and OPCNF a larger specific surface area. As shown in Fig. S3 and Table S1, the Brunauer–Emmett–Teller surface area of PCNF and N-PCNF are 285.4 and 353.5 m^2^ g^−1^, respectively. After exfoliation by concentrated nitric acid, the specific surface area of finally OPCNF and N-OPCNF increased to 543.5 and 570.4 m^2^ g^−1^, respectively. It should be noted that the desorption branch and adsorption branch of the isotherm are not closed in the low-pressure area, which may be due to the existence of ink-bottle pores and restricted-access pores [29, 30]. Therefore, in-situ exfoliating of carbon nanosheets can make N-OPCNF have more structural defects and a larger specific surface area, so as to provide more sites for the adsorption of electrolyte ions.

The surface chemical composition of four samples was studied by X-ray photoelectron spectroscopy (XPS). As shown in Fig. S4, the addition of melamine as a nitrogen source and subsequent nitric acid oxidation successfully introduced a high proportion of nitrogen (3.71 at%) and oxygen (13.75 at%) into N-OPCNF. The high-resolution N 1*s* spectrum of N-PCNF and N-OPCNF (Fig. 1h) showed four typical peaks centered at 398.3, 399.9, 401.3, and 403.5 eV, indicating the presence of pyridine N (N6), pyrrole N (N5), graphite N (NQ) and oxide N species, respectively [31, 32]. After 5 h of oxidation in concentrated nitric acid, a new peak centered at 405.7 eV appears in N-OPCNF, which is assigned to NO_x_ [33]. In addition, nitric acid treatment increased the N5 content (Table S2) from 18.22% of N-PCNF to 39.24% of N-OPCNF, and the corresponding NQ content decreased from 48.88% of N-PCNF to 26.47% of N-OPCNF. The same phenomenon occurs in PCNF and OPCNF (Fig. S5a), where a small amount of N element comes from the decomposition of PAN. The high-resolution O 1*s* spectrum (Fig. 1i) of N-PCNF can be fitted to carbonyl, alcohol and carboxyl groups [34]. After oxidation by nitric acid, the content of carbonyl groups increased from 19.55% of N-PCNF to 52.6% of N-OPCNF, and the proportion of corresponding alcohol groups decreased from 69.97% to 36.68%. The same phenomenon also occurs in PCNF and OPCNF (Fig. S5b). These results show that nitric acid oxidation reduces the relative concentration of alcohol groups on the surface of carbon nanofibers and promotes the formation of carbonyl groups [35, 36]. Since carbonyl oxygen is the most effective electron absorbing group among the three oxygen groups, the increase of the proportion of carbonyl makes part of the electron density of alcohol group taken away by carbonyl oxygen, which leads to the whole O 1*s* peak position of N-OPCNF moving to a higher binding energy [37]. This phenomenon was also observed from the Fourier transformed infrared spectra (Fig. S6). After nitric acid oxidation, the adsorption peak corresponding to C=O (1729 cm^−1^) was significantly enhanced in the spectra of OPCNF and N-OPCNF, indicating the formation of carbonyl groups [38]. Benefiting from the introduction of nitrogen-doped and carbonyl-functionalized surface, the water contact angle (Figs. 1j and S7) of the N-OPCNF is significantly smaller than other samples (reduced from 153.4° to 32.7°). Overall, the additional nitrogen additives and nitric acid exfoliating treatment create a hydrophilic, high pyridine/pyrrole nitrogen doped, and carbonyl-functionalized surface for N-OPCNF.

### Electrochemical Performance of Aqueous ZIHCs

**Fig. 2:**
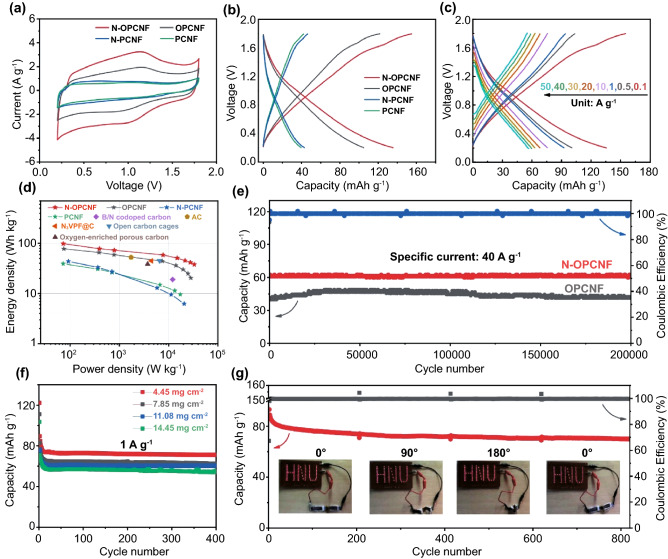
**a** CV curves of PCNF, N-PCNF, OPCNF and N-OPCNF at a scan rate of 5 mV s^−1^ **b** GCD profiles of PCNF, N-PCNF, OPCNF and N-OPCNF at 0.1 A g^−1^ **c** GCD profiles of N-OPCNF at different current densities. **d** Ragone plots of N-OPCNF based ZIHCs compared with previously reported ZIHCs. **e** Long-term cyclic stability of N-OPCNF at 40 A g^−1^. **f** Cycling performance of N-OPCNF under various mass loadings at 1 A g^−1^. **g** Cycling performance of N-OPCNF pouch cell under different bending angles at 2 A g^−1^ (Inset: The N-OPCNF pouch cell lights up an “HNU” Logo in the different bent states)

In order to evaluate the electrochemical performance of the prepared carbon nanofibers film as a flexible cathode of ZIHCs, aqueous ZIHCs were assembled by using self-supporting carbon nanofibers film as cathode (Fig. S8) and Zn foil as anode in 1 M ZnSO_4_ solution. Figure 2a shows the cyclic voltammetry (CV) curves of different carbon nanofibers film cathodes at a scan rate of 5 mV s^−1^ in the voltage range of 0.2 to 1.8 V. The ZIHCs with PCNF and N-PCNF working electrodes have nearly rectangular CV curves representing the ideal EDLC [39, 40]. Compared with PCNF, the CV curve of N-PCNF does not change significantly, indicating that a single nitrogen dopant is not enough to improve the adsorption of electrolyte ions on the carbon nanofibers cathode. After exfoliating carbonyl-functionalized nanosheets by nitric acid treatment, a set of symmetrical Faradaic charge/discharge peaks appeared on the CV curve of OPCNF, indicating that the carbonyl groups have pseudocapacitive activity. This kind of pseudocapacitance is generally considered to be derived from the chemisorption between Zn^2+^ and carbonyl groups [15, 41, 42]. With the simultaneous introduction of carbonyl groups and pyridine/pyrrole nitrogen dopants on the surface of carbon nanofibers, the peak intensity and area of the CV curve of N-OPCNF electrode further increase, indicating that there is a synergistic effect between the pyridine/pyrrole nitrogen dopants and the carbonyl groups to promote the enhancement of the pseudocapacitance effect. The galvanostatic charge/discharge (GCD) curves of all samples at 0.1 A g^−1^ are shown in Fig. 2b. The reversible capacity of N-OPCNF (136 mAh g^−1^) is higher than that of OPCNF (105 mAh g^−1^), N-PCNF (43 mAh g^−1^) and PCNF (40 mAh g^−1^), which is consistent with the CV test results. Similarly, N-OPCNF also has the best rate performance at different current densities from 0.1 to 50 A g^−1^ (Fig. 2c). The average reversible capacities of N-OPCNF are 136, 101, 93, 76, 68, 63, and 60 mAh g^−1^ at 0.1, 0.5, 1, 10, 20, 30, and 40 A g^−1^, respectively. Even at an ultra-high current density of 50 A g^−1^, N-OPCNF still maintain a high capacity of 57 mAh g^−1^, much higher than OPCNF, N-PCNF and PCNF (Fig. S9). Considering that nitric acid oxidation will destroy the π-conjugated network of carbon nanofibers [43], resulting in the decrease of electrode conductivity (Fig. S10 and Table S3). The performance of the oxidized sample is significantly higher than that of the unoxidized sample, which indicates that the pseudocapacitive contribution of the oxygen functional group is much higher than its effect on the electrode conductance.

It is worth noting that the specific surface area of carbon nanofibers is also improved after the introduction of carbonyl groups by nitric acid oxidation. In order to evaluate the different contributions of the introduction of carbonyl groups and the increase of specific surface area to the capacitance of the ZIHCs, we prepared pure carbon nanofibers (labeled as CNF) by electrospinning a precursor solution containing polyacrylonitrile and DMF and using the same annealing conditions. After 5 h of nitric acid oxidation treatment (the resulting sample is labeled OCNF), the specific surface area of CNF increased from 16.5 to 139.2 m^2^ g^−1^ (Fig. S11a-b). At the same oxidation level (5 h), the difference of specific surface area between OPCNF and OCNF was 404.3 m^2^ g^−1^. As a result, the capacity difference between OPCNF and OCNF at 0.1 A g^−1^ is 28.5 mAh g^−1^ (Fig. S12b). In contrast, although the specific surface area difference between PCNF and OCNF is 146.2 m^2^ g^−1^, the capacity difference between PCNF and OCNF is 36.8 mAh g^−1^ due to the absence of pseudocapacitance contribution caused by carbonyl groups. Therefore, the capacity gain brought by the introduction of carbonyl groups on the carbon nanofibers is much higher than the gain brought about by the increase in the specific surface area of the carbon nanofibers. More encouragingly, N-OPCNF delivers a maximum energy density of 98.28 Wh kg^−1^ at a power density of 72.27 W kg^−1^ (Fig. 2d) and still retains an energy density of 42 Wh kg^−1^ even at a high power density output of 33.2 kW kg^−1^ (based on the mass of the N-OPCNF electrode), which is better than previously reported Zn-ion batteries and ZIHCs [44–47]. Combined with the surface area and XPS characterization of above samples, it is suggested that the exfoliation of the high pyridine/pyrrole nitrogen-doped and carbonyl-functionalized nanosheets with high specific surface area on the carbon nanofibers cathode can optimize the pseudocapacitance of ZIHCs and further increase its energy density to a higher level.

In addition to excellent energy density, both N-OPCNF and OPCNF electrodes exhibited ultra-long cycling lifespan after 200,000 cycles at 40 A g^−1^ (Fig. 2e), with capacity retention of 99.2% and 97.3%, respectively, which is significantly better than previous reports (Table S4). The performance stability mechanism of the N-OPCNF electrode was studied by electrochemical impedance spectroscopy and SEM. As shown in Fig. S13a, even after 200,000 cycles, the charge transfer resistance (*R*_*ct*_) of N-OPCNF changes only slightly, while the electrolyte diffusion resistance in the low-frequency region remains almost stable, indicating that the N-OPCNF electrode has a stable electrode/electrolyte interface and ion migration path. In addition, compared with the initial N-OPCNF electrode (Fig. S14a), the N-OPCNF electrode after cycling (Fig. S14b) also showed a complete carbon nanofiber structure, indicating excellent structural stability of carbon nanofibers. It is worth noting that two separators were used in the long cycle test to prevent short circuit of the battery caused by growth of zinc dendrites (Fig. S14c-d). In order to further evaluate the performance of N-OPCNF electrode in practical application, the performance tests were carried out for the electrodes with mass loads of 4.45, 7.85, 11.08, and 14.45 mg cm^−2^, respectively. As shown in Fig. 2f, N-OPCNF maintains good capacity and cycle stability even under an ultra-high mass load of 14.45 mg cm^−2^, which indicates that N-OPCNF has practical application prospects. The good mass loading capacity of the N-OPCNF electrode may be derived from the optimized *R*_*ct*_ (Fig. S13b) and improved electrolyte surface wettability (Fig. 1j) owing to the introduction of nitrogen dopants and carbonyl groups, which promote the electrolyte ion and electron transportation. In addition to excellent high load capacity, N-OPCNF electrodes also have flexible application prospects. As shown in Fig. 2g, the pouch battery has good cycle stability under different bending angles from 0° to 180° at the current density of 2 A g^−1^. In addition, the GCD curves of the pouch battery under different bending angles approximately overlap (Fig. S15), which confirms its good flexibility. The illustration in Fig. 2g shows that two series connected pouch batteries can supply power to the "HNU" array containing 75 light-emitting diodes under different bending states, which provides a reference for commercial applications of flexible ZIHCs.

### Energy Storage Mechanism of Aqueous ZIHCs

**Fig. 3:**
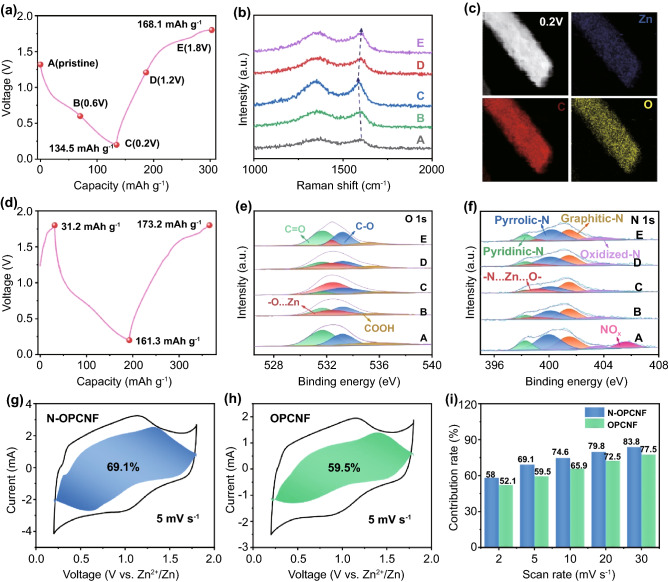
**a** The first GCD profile in the mode of discharge first and charge at 0.1 A g^−1^. **b** Ex-situ Raman spectra, **c** EDS mappings at full discharged state, **d** GCD profiles in the mode of charge first and discharge at 0.1 A g^−1^. **e** Ex-situ O 1*s* spectra. **f** Ex-situ N 1*s* spectra. **g** Capacitive contribution at 5 mV s^−1^ of N-OPCNF. **h** Capacitive contribution at 5 mV s^−1^ of OPCNF. **i** Capacitive contribution ratio comparison at different scan rates for N-OPCNF and OPCNF

In order to explain why N-OPCNF has the best performance for ZIHCs, we tried to explore the energy storage mechanism of N-OPCNF electrode. As showed in Fig. 3a, five representative moments (A, B, C, D, and E) are selected on the first GCD curve of N-OPCNF electrode to track its surface composition and morphology change. The ex-situ Raman spectrum in Fig. 3b shows that when the fresh N-OPCNF electrode is discharged from the open circuit potential (1.32 V) to 0.2 V, the intensity of the D band and G band of the Raman spectrum gradually increase. This phenomenon is attributed to the suppression of phonon energy dissipation by the adsorption of Zn^2+^ on the N-OPCNF electrode [13]. In addition, the G-band of N-OPCNF in the fully discharged state exhibits a red shift, which should also be attributed to the interaction between the adsorbed Zn^2+^ and the N-OPCNF cathode [48]. The TEM element mapping (Fig. 3c) also shows that Zn elements are evenly distributed on the electrode in the fully discharged state, which further proves that Zn^2+^ is successfully adsorbed on the electrode. When the N-OPCNF electrode is charged from 0.2 to 1.2 V, the intensity of Raman spectrum decreases gradually, indicating the desorption of Zn^2+^. As the N-OPCNF electrode is further charged to 1.8 V, the intensity of the Raman spectrum increases again, which is mainly due to the adsorption of SO_4_^2−^ by the electrode during the charging process [11]. The adsorption of anions in the electrolyte by the N-OPCNF electrode in the fully charged state also resulted in the incomplete recovery of the red shift of the G-band after full charging. Due to the coexistence of the desorption of Zn^2+^ and the adsorption of SO_4_^2−^ during the charging process, the first charging capacity (168.1 mAh g^−1^) in the GCD curve (Fig. 3a) is 33.6 mAh g^−1^ higher than the first discharge capacity (134.5 mAh g^−1^), which results in the initial Coulomb efficiency exceeding 100%. In contrast, when the fresh N-OPCNF electrode was first charged from the open circuit potential to 1.8 V (Fig. 3d), the electrode obtained a capacity of 31.2 mAh g^−1^ due to the adsorption of SO_4_^2−^. In the next discharge/charge cycle, due to the coexistence of adsorption/desorption of Zn^2+^ and desorption/adsorption of SO_4_^2−^, the difference between the charging capacity (173.2 mAh g^−1^) and discharge capacity (161.3 mAh g^−1^) of the N-OPCNF electrode is significantly reduced. Therefore, the total capacitance of N-OPCNF electrodes is mainly contributed by the pseudocapacitance generated by adsorbed Zn^2+^, while the capacitance contribution of typical EDLCs is relatively low.

In order to obtain the details about the chemical conversion of the electrode during the charging and discharging process, the ex-situ XPS spectra have been studied. Figure 3e displayed the ex-situ O 1*s* spectra of the N-OPCNF electrode. During the discharge, the ratio of the characteristic peaks corresponding to the carbonyl group (531.3 eV) decreased continuously, and a new peak at 532.1 eV corresponding to the C–O–Zn bond was detected [15, 49]. During the charging process, a reversible increase in the ratio of carbonyl groups was observed, accompanied by the gradual disappearance of the C–O–Zn peak, which indicated that a reversible pseudocapacitance reaction occurred between the Zn^2+^ and the carbonyl groups on the surface of the carbon nanofibers [41, 50]. Moreover, it can be observed from the ex-situ N 1*s* spectrum (Fig. 3f) that the ratio of pyridine/pyrrole nitrogen gradually decreases with the progress of the discharge process, and a new peak corresponding to the N-Zn–O bond is detected at 399.2 eV [51]. This result indicates that the pyridine/pyrrole nitrogen dopants can promote the adsorption of Zn^2+^ by bonding with the carbonyl group to form N-Zn–O bond. During the subsequent charging process, it can be observed that the ratio of pyridine/pyrrole nitrogen increases reversibly, indicating the removal of Zn^2+^ from the N-Zn–O bond. Thus, the N-OPCNF cathodes with multi-chemisorption sites of pyridine/pyrrole nitrogen atoms and carbonyl groups possess a new chemisorption mechanism compared with the previously reported carbon-based ZIHCs [12, 15, 23, 52–54].

The ex-situ tests described above and the CV analysis of Fig. 2a have jointly proved that reversible pseudocapacitance reactions occur between the carbonyl groups and Zn^2+^, and pyridine/pyrrole nitrogen dopants can further increase this pseudocapacitance reaction by bonding with the carbonyl group to form N–Zn–O bond. In order to quantitatively describe the effect of pyridine/pyrrole nitrogen doping on the capacitive behavior of the electrode, CV analyses of N-OPCNF and OPCNF electrodes were carried out at scan rates from 2 to 30 mV s^−1^ (Fig. S16). Based on Dunn's method, the surface process contribution (*k*_*1*_*v*) at a fixed scanning rate can be quantified by Eq. (1) [55]:

1 $$i\left( V \right) = \, k_{{1}} v \, + \, k_{{2}} v^{{{1}/{2}}}$$

As shown in Fig. 3g, at the scanning rate of 5 mv s^−1^, nearly 69.1% of the total storage charge of N-OPCNF is contributed by capacitive charge (blue region), which is higher than 59.5% of OPCNF (Fig. 3h). In addition, the pseudocapacitance contribution ratio of N-OPCNF gradually increases with the increase of the scan rate, and finally reaches the maximum value of 83.8% at 30 mV s^−1^ (Fig. 3i). The above results confirm that the charge storage method of N-OPCNF is mainly a pseudocapacitance process, and reveal that pyridine/pyrrole nitrogen doping can increase the contribution of pseudocapacitance.

**Fig. 4:**
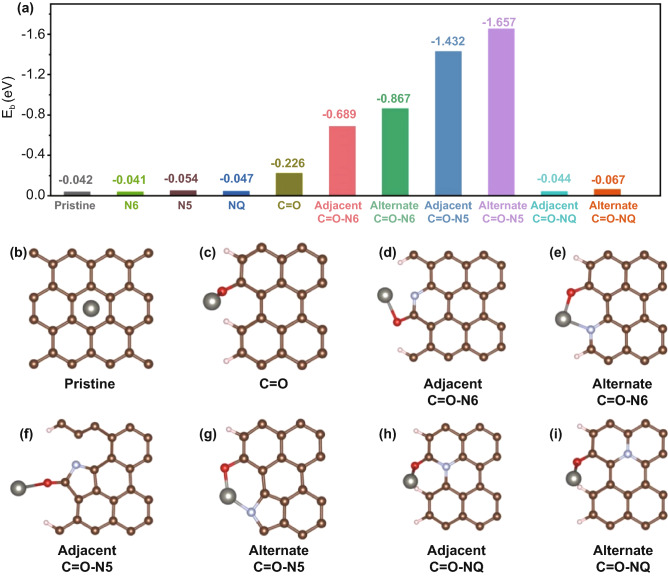
**a** The binding energies of Zn atom on pristine graphene and different functionalized graphene. Optimized structures of Zn adsorption on the surfaces of **b** ideal graphene, **c** carbonyl functionalized graphene, **d** adjacent N6-doped and carbonyl functionalized graphene, **e** alternate site N6-doped and carbonyl functionalized graphene **f** adjacent N5-doped and carbonyl functionalized graphene, **g** alternate site N5-doped and carbonyl functionalized graphene, **h** adjacent NQ-doped and carbonyl functionalized graphene, and **i** alternate site NQ-doped and carbonyl functionalized graphene. Brown, white, red, and gray balls represent C, H, O, and Zn atoms

In order to reveal how the carbonyl groups and N dopants affect the adsorption of Zn^2+^ on the surface of carbon cathode, the adsorption between Zn^2+^ and different functionalized structures were theoretically studied based on density functional theory (DFT) calculation, and the corresponding binding energies (Δ*E*_b_) are plotted in Fig. 4a. The calculation results suggest that the Δ*E*_b_ between pristine graphene and Zn^2+^ was only −0.042 eV (Fig. 4b), which indicates that the adsorption of Zn^2+^ on the surface of pristine graphene was weak. The optimized Zn atom adsorption configuration is located on the top of the six-membered carbon ring, and there is no bonding property. After introducing a carbonyl group on the graphene edge, the Δ*E*_b_ of bonding to form C–O–Zn bond was calculated to be −0.226 eV (Fig. 4c), which indicated that chemical adsorption occurs between the carbonyl group and Zn^2+^. However, when different N-doping sites (N6, N5, and NQ) were introduced into graphene separately, the corresponding Δ*E*_b_ were calculated to be −0.041, −0.054, and −0.047 eV, respectively (Fig. S17a-c), which indicated that independent N-doping had little effect on the adsorption of Zn^2+^ on graphene surface. Encouragingly, the adsorption of Zn^2+^ was significantly enhanced by simultaneously introducing a carbonyl group and N6 or N5 atom into graphene surface. When the doped N6 or N5 atom was adjacent to the carbonyl group, the Δ*E*_b_ of bonding to form C–O–Zn bond reached −0.689 and −1.432 eV, respectively (Fig. 4d, f), indicating that N6 and N5 dopants can effectively improve the chemisorption of carbonyl group to Zn^2+^. In order to understand this phenomenon, bader charge analysis was used to describe the effect of N6 and N5 atoms on the electron states of neighboring atoms. Specifically, after introducing N6 and N5 atoms into graphene, the electrons of carbon atoms adjacent to N6 and N5 atoms will be partially transferred to N6 and N5 atoms with higher electronegativity to buffer the strong electron affinity of N6 and N5 atoms [56]. Figure S18b-c showed that the charges of 1.125 and 1.182 were transferred from adjacent carbon atoms to N6 and N5 atoms in the graphene structures containing carbonyl and adjacent N6 or N5 atoms, respectively. At this time, the carbon atom adjacent to the N6 and N5 dopant has a higher positive charge density. As shown in Fig. S18b-c, in the structure containing the adjacent N6 or N5 atom, the positive charge of the carbon atom in the carbonyl group are 1.264 and 1.331, respectively, which are much greater than 0.731 of the structure functionalized with only the carbonyl group (Fig. S18a). The resulting charge delocalization effect leads to stronger polarization of carbonyl covalent bond in the graphene structure with adjacent N6 or N5 atoms than that of the structure functionalized with only the carbonyl group [15]. Therefore, the adsorption of Zn^2+^ through the fracture of carbonyl bond is more likely to occur on adjacent N6 or N5 doped carbon materials. In addition, when the doped N6 or N5 atom and carbonyl group are distributed in alternate positions (Fig. 4e, g), the Δ*E*_b_ of bonding to form N6-Zn–O and N5-Zn–O bonds can reach −0.867 and −1.657 eV, respectively. Through modeling, the bond lengths of N6-Zn and N5-Zn are 2.07 and 1.84 Å, respectively, indicating strong chemical bonding. The electron density difference of Zn adsorption indicates that the charge transferred from the Zn atom to the carbonyl-functionalized structure is only 0.14 (Fig. S19a), and most of the electrons accumulate on the top of the Zn atom. In contrast, Zn atoms transfer 0.61 and 1.12 charges to the structures containing alternate N6 (Fig. S19b) and N5 (Fig. S19c) sites, respectively. This finding further confirmed that the simultaneous introduction of pyridine/pyrrole nitrogen dopants and carbonyl groups promoted the chemisorption between graphene and Zn^2+^. The relatively low Δ*E*_b_ (Fig. 4f, i) of the NQ site may be derived from its saturated electron orbital [57], which has a negligible effect on Zn^2+^ adsorption [58]. In summary, the effects of pyridine/pyrrole nitrogen dopants and carbonyl groups on the adsorption of Zn^2+^ on the surface of carbon cathodes can be divided into two aspects: First, the pyridine/pyrrole nitrogen atoms adjacent to the carbonyl groups can greatly reduce the binding energy between carbonyl groups and Zn^2+^ by inducing charge delocalization of carbonyl groups. Secondly, the carbonyl groups can further chemically adsorb Zn^2+^ by bonding with the alternate pyridine/pyrrole nitrogen atoms to form N-Zn–O bond. These results theoretically verify the enhanced Zn^2+^ storage capacity of N-OPCNF.

### Electrochemical Performance of Quasi-Solid ZIHCs

**Fig. 5:**
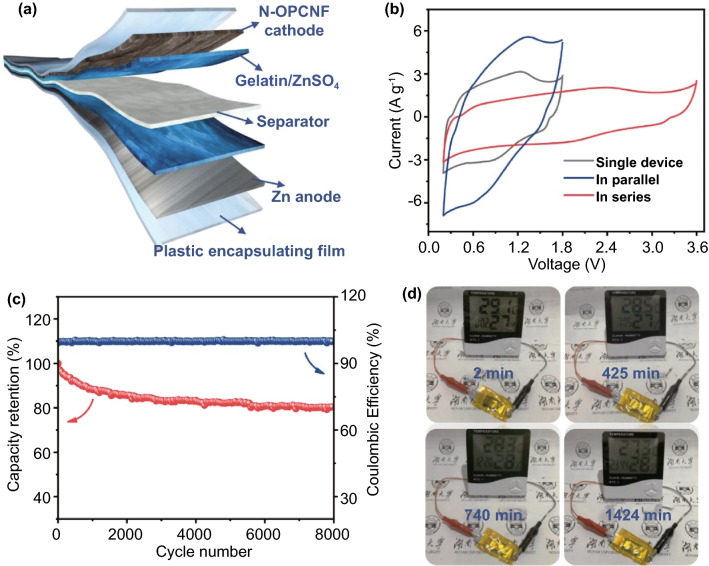
**a** Schematic diagram of the as-assembled quasi-solid-state ZIHC. **b** CV profiles of single device, two devices in parallel and in series at 10 mV s^−1^. **c** Capacitance retention and Coulombic efficiency of the quasi-solid-state ZIHC at 1 A g^−1^ for 8000 cycles. **d** Photograph records of a quasi-solid-state ZIHC powered timer at different discharge times

In order to further exemplify the feasibility application of N-OPCNF as a cathode of ZIHC, as shown in the configuration of Fig. 5a, we assembled a quasi-solid device using gelatin electrolyte (Fig. S20). The ionic conductivity of the gelatin electrolyte was calculated to be 3.67 ms cm^−1^ based on the method in Fig. S21 [59, 60]. Figure 5b shows the CV test results under different conditions in simulated actual application. Specifically, the overall capacity can be doubled when two devices are connected in parallel. Moreover, integrating two devices connected in series can provide double voltage (3.6 V). In addition, the assembled quasi-solid ZIHC has a long cycle life, with a capacity retention rate of 80.38% after 8000 cycles at 1 A g^−1^ (Fig. 5c). Finally, the assembled single quasi-solid ZIHC is used to power the electronic timer, which has a working time of more than 23 h (Fig. 5d).

## Conclusions

In conclusion, we have successfully modified multiple chemisorption sites on carbon nanofibers by in-situ exfoliating of high pyridine/pyrrole nitrogen-doped and carbonyl-functionalized nanosheets. The obtained flexible self-supporting N-OPCNF cathode integrates the advantages of large specific surface area, strong hydrophilicity, and multi-chemisorption sites with high Zn^2+^ adsorption activity. Ex-situ analyses and DFT calculations show that both carbonyl groups and pyridine/pyrrole nitrogen atoms can serve as the Zn^2+^ host during the discharging process, while the highly electronegative pyridine/pyrrole nitrogen atoms can also act as a strong electron acceptor to induce the charge delocalization of the carbonyl, so as to improve the Zn^2+^ adsorption activity of carbonyl groups. Hence, the N-OPCNF cathodes with multi-chemisorption sites show extremely high energy/power density (98.28 Wh kg^−1^ and 33.2 kW kg^−1^) and ultra-long-term cycle stability (remaining 99.2% of initial capacity at 40 A g^−1^ after 200,000 cycles). More importantly, the N-OPCNF cathode delivers a high capacitance retention rate (66.3% of the initial value) even under an ultra-high mass load of 14.45 mg cm^−2^. The surface engineering strategy proposed in this work effectively solves the bottleneck problem of carbon-based ZIHC lacking active Zn^2+^ adsorption sites, and may be extended to the development of other high-performance energy storage devices.

## Supplementary Information

Below is the link to the electronic supplementary material.

Supplementary file1 (PDF 1603 KB)
