# Supplementary material for: Enabling Multi-Chemisorption Sites on Carbon Nanofibers Cathodes by an In-situ Exfoliation Strategy for High-Performance Zn–Ion Hybrid Capacitors
Source: Nanomicro Lett. 2022 Apr 15;14:106. doi: 10.1007/s40820-022-00839-z (PMC9012804; doi:10.1007/s40820-022-00839-z)
Supplement: Supplementary file 1 — Supplementary file1 (PDF 1603 KB) [file 40820_2022_839_MOESM1_ESM.pdf]

Supporting Information for

Enabling Multi-Chemisorption Sites on Carbon Nanofibers Cathodes by an In-Situ Exfoliation Strategy for High-Performance Zn-Ion Hybrid Capacitors

Hongcheng He[1], Jichun Lian[1], Changmiao Chen[2], Qiaotian Xiong[1], Cheng Chao Li[3, *], and Ming Zhang[1, *]

[1] Key Laboratory for Micro/Nano Optoelectronic Devices of Ministry of Education, Hunan Provincial Key Laboratory of Low-Dimensional Structural Physics & Devices, Hunan Joint International Laboratory of Advanced Materials and Technology for Clean Energy, School of Physics and Electronics, College of Semiconductors (College of Integrated Circuits), Hunan University, Changsha 410082, P. R. China

[2] Tsinghua Shenzhen International Graduate School, Tsinghua University, Shenzhen 518055, P. R. China.

[3] School of Chemical Engineering and Light Industry, Guangdong University of Technology, Guangzhou 510006, P. R. China

*Corresponding authors. E-mail: zhangming@hnu.edu.cn (Ming Zhang); licc@gdut.edu.cn (Cheng Chao Li)

Supplementary Figures and Tables

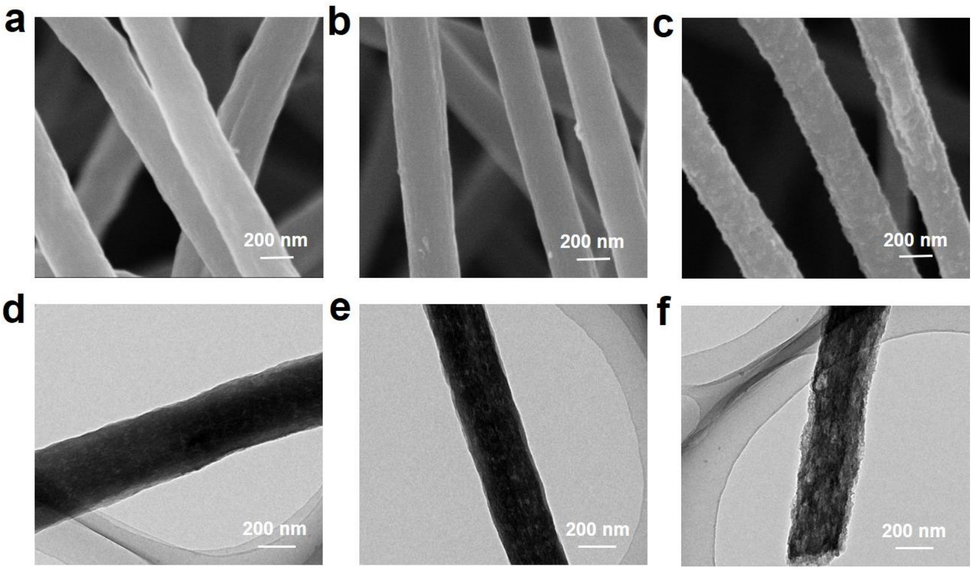

Fig. S1 (**a**) SEM image of PCNF. (**b**) SEM image of N-PCNF. (**c**) SEM image of OPCNF. (**d**) TEM image of PCNF. (**e**) TEM image of N-PCNF. (**f**) TEM image of OPCNF

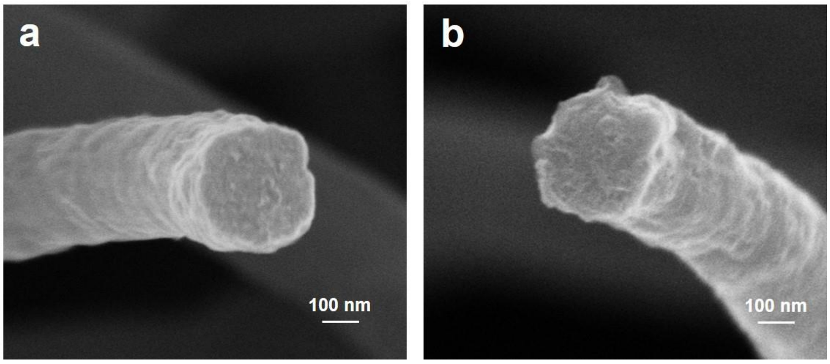

Fig. S2 cross-sectional SEM images of (**a**) N-PCNF and (**b**) N-OPCNF

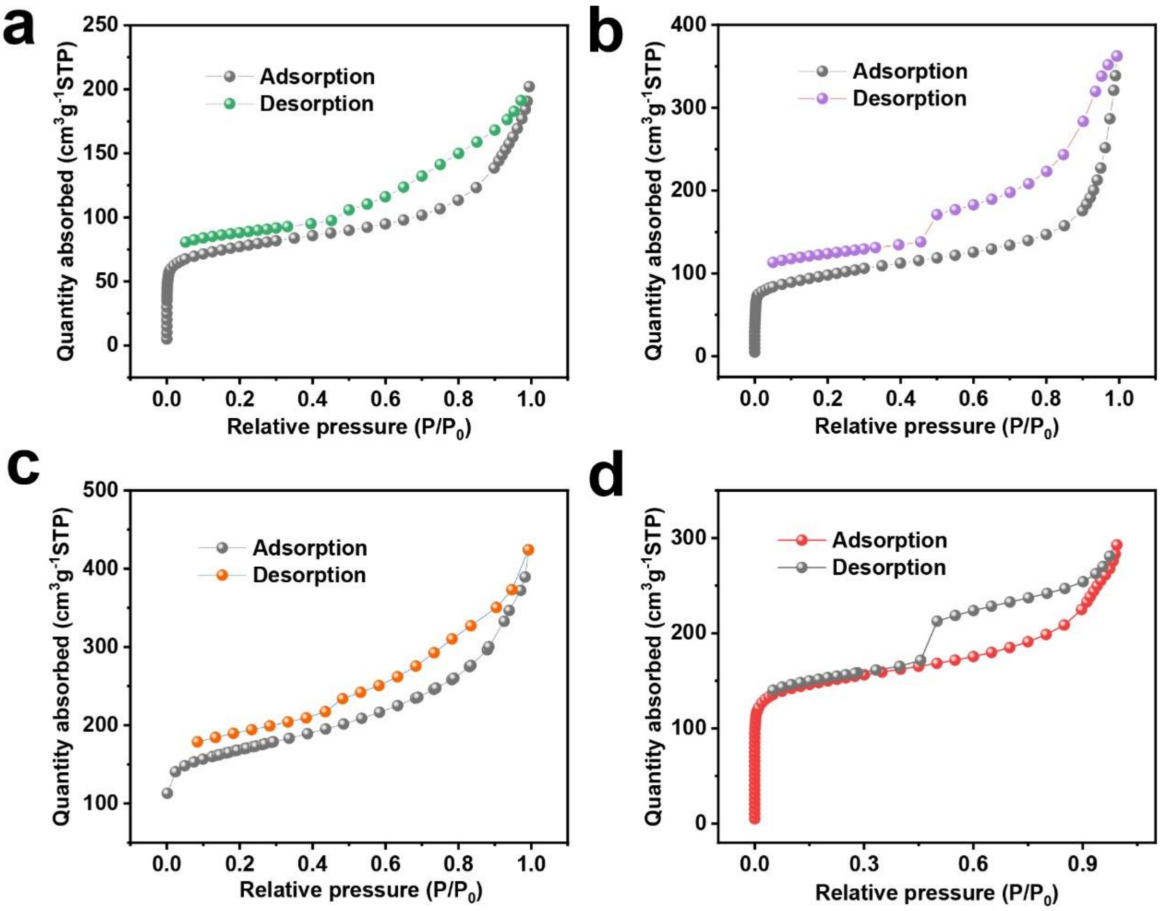

Fig. S3 Nitrogen adsorption-desorption isotherms curve of (**a**) PCNF, (**b**) N-PCNF, (**c**) OPCNF and (**d**) N-OPCNF

Table S1 Comparison of the specific surface area of different samples based on BET tests

	**PCNF**	**N-PCNF**	**OPCNF**	**N-OPCNF**
**surface area (m^2 g^{-1})**	285.4	353.5	543.5	570.4

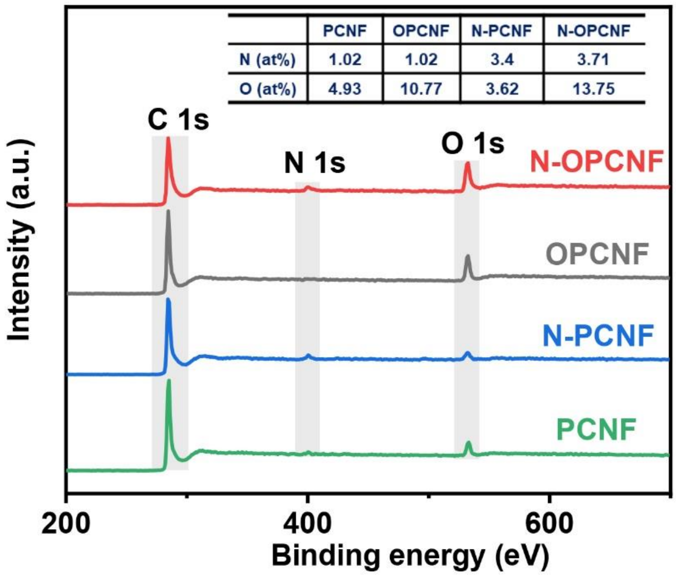

Fig. S4 XPS survey spectrum of N-OPCNF, OPCNF, N-PCNF and PCNF

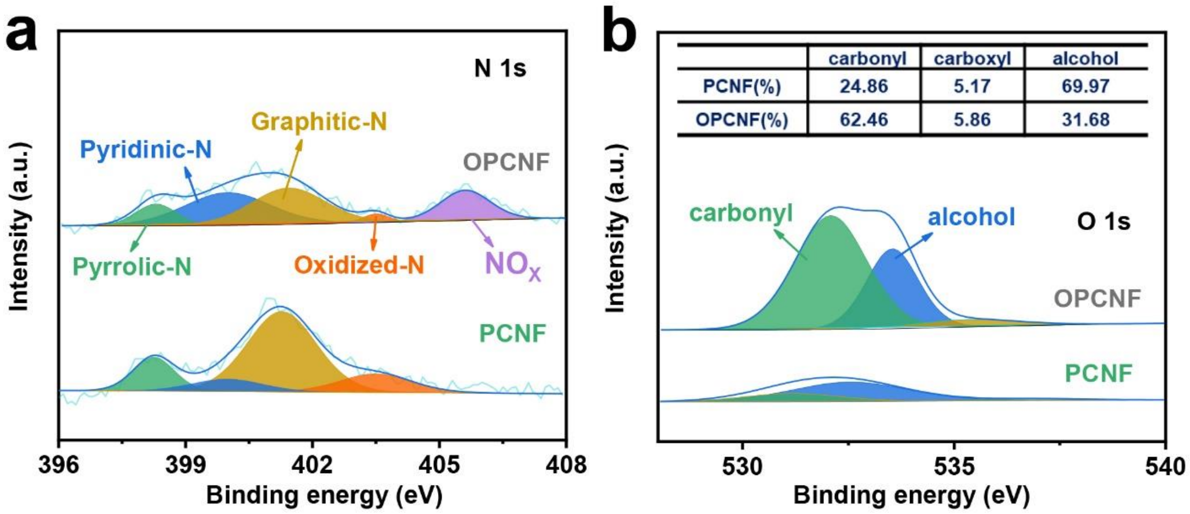

Fig. S5 (**a**) High-resolution N 1s and (**b**) O 1s XPS spectra of PCNF and OPCNF

Table S2 Comparing the relative concentrations of the nitrogen functional groups at different nitric acid treatment times based on XPS tests

Materials	Pyridine N	Pyrrole N	Graphite N	Oxide N	NOx
**PCNF**	15.65	3.76	67.23	13.35	0
**OPCNF**	10.49	33.52	32.33	2.4	21.26
**N-PCNF**	17.44	18.22	48.88	15.46	0
**N-OPCNF**	14.23	39.24	26.47	8.44	11.61

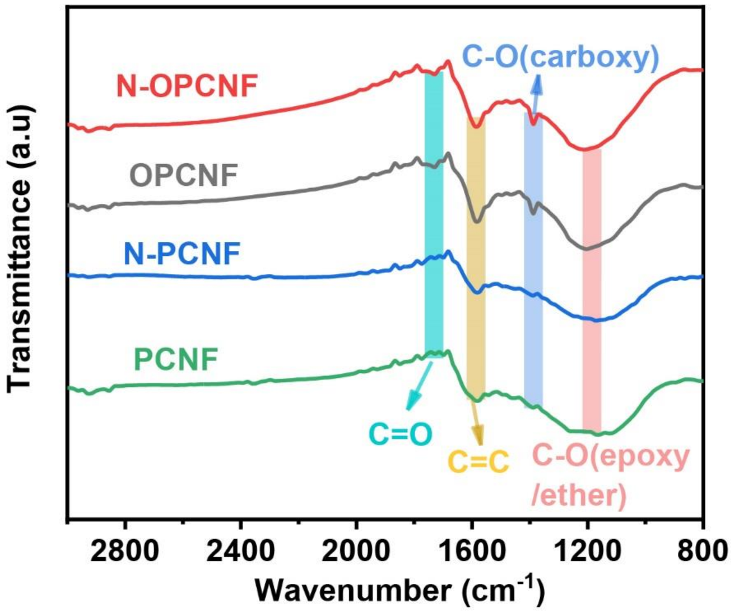

Fig. S6 FTIR spectra of N-OPCNF, OPCNF, N-PCNF and PCNF

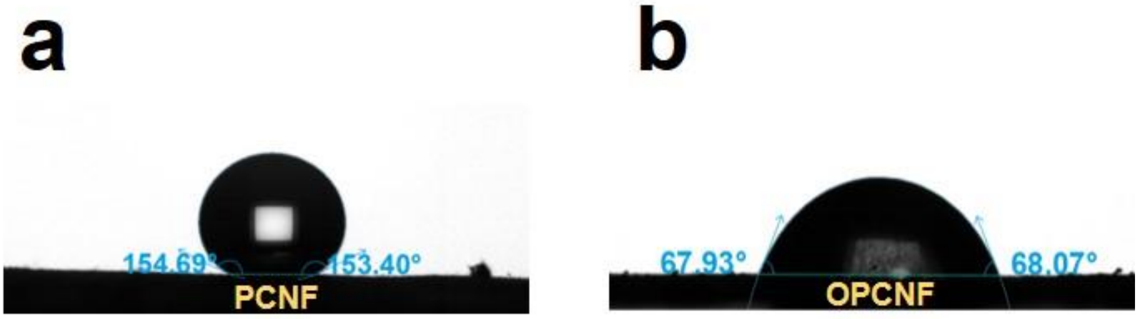

Fig. S7 Contact angles of a water droplet on (**a**) PCNF and (**b**) OPCNF

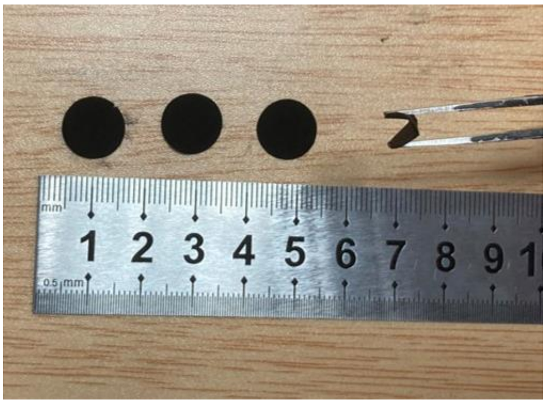

Fig. S8 The flexible N-OPCNF electrode

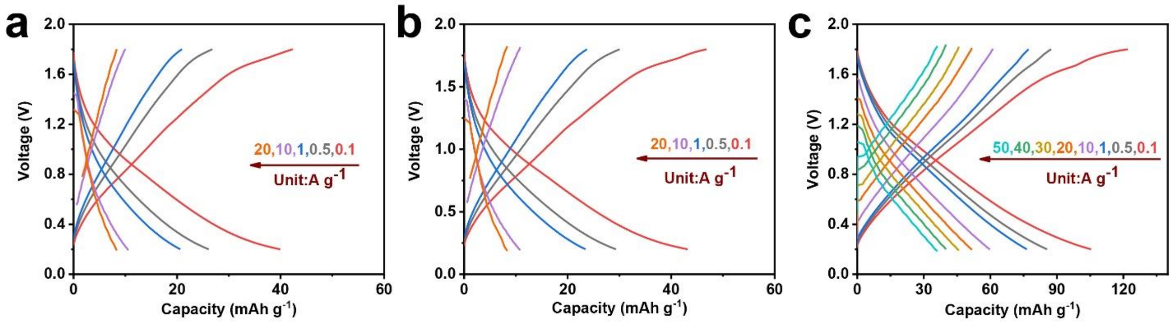

Fig. S9 GCD profiles of (**a**) PCNF, (**b**) N-PCNF and (**c**) OPCNF

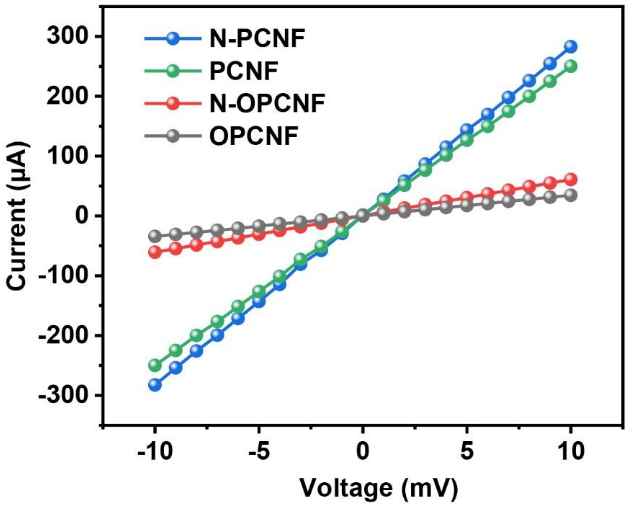

Fig. S10 *I*–*V* test of N-PCNF, PCNF, N-OPCNF and OPCNF using the fibrofelt

Table S3 Comparing the conductance of N-PCNF, PCNF, N-OPCNF and OPCNF based on I–V tests

Materials	N-PCNF	PCNF	N-OPCNF	OPCNF
**Conductance (S m^{-1})**	2.83×10^{-2}	2.51×10^{-2}	0.61×10^{-2}	0.35×10^{-2}

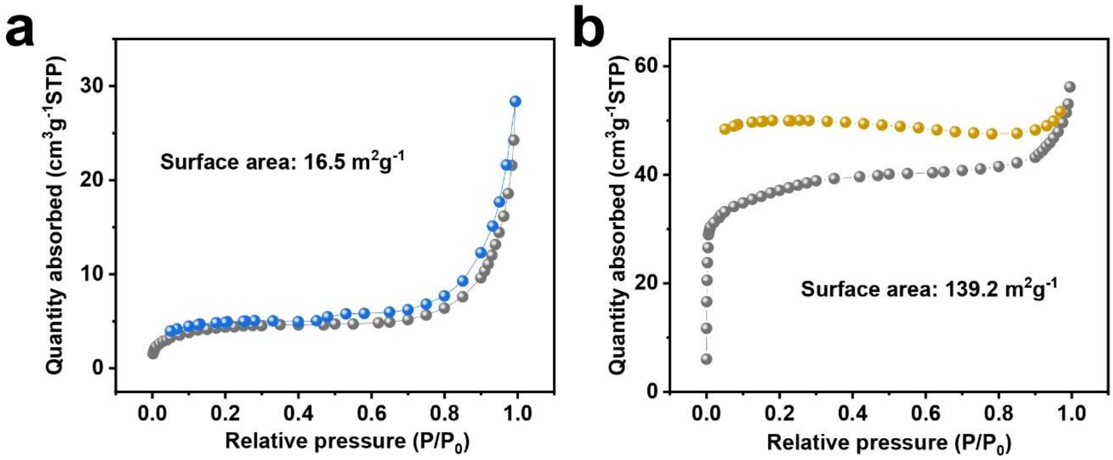

Fig. S11 Nitrogen adsorption-desorption isotherms curve of (**a**) CNF and (**b**) OCNF

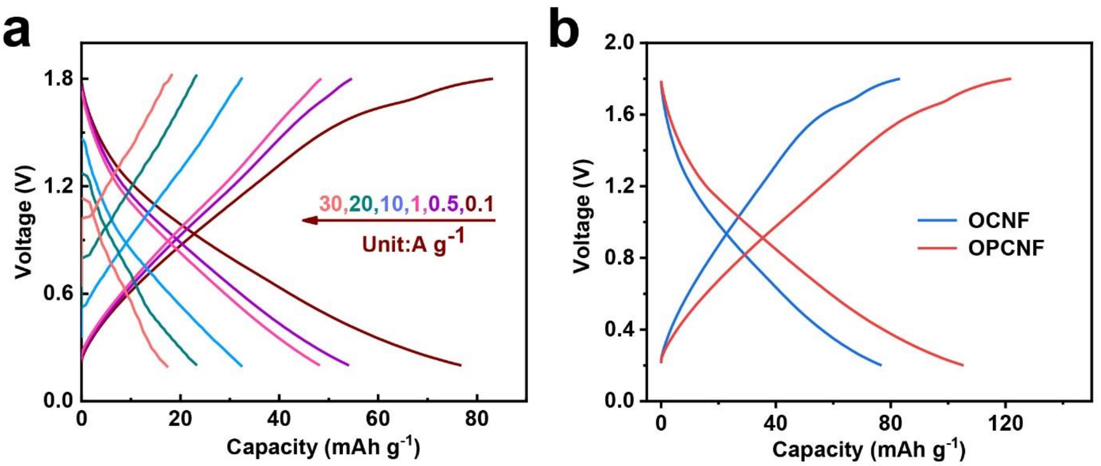

Fig. S12 (**a**) GCD profiles of OCNF at different current densities. (**b**) GCD profiles of OCNF OPCNF at 0.1 A g^{-1}

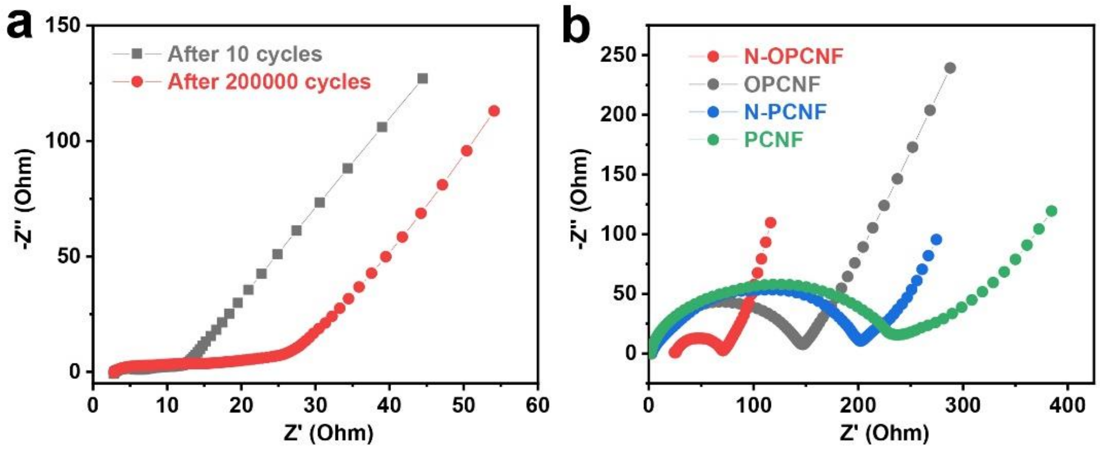

Fig. S13 Nyquist plots of (**a**) N-OPCNF electrode at different cycles at 40 A g^{-1}. (**b**) N-OPCNF, OPCNF, N-PCNF and PCNF before cycle

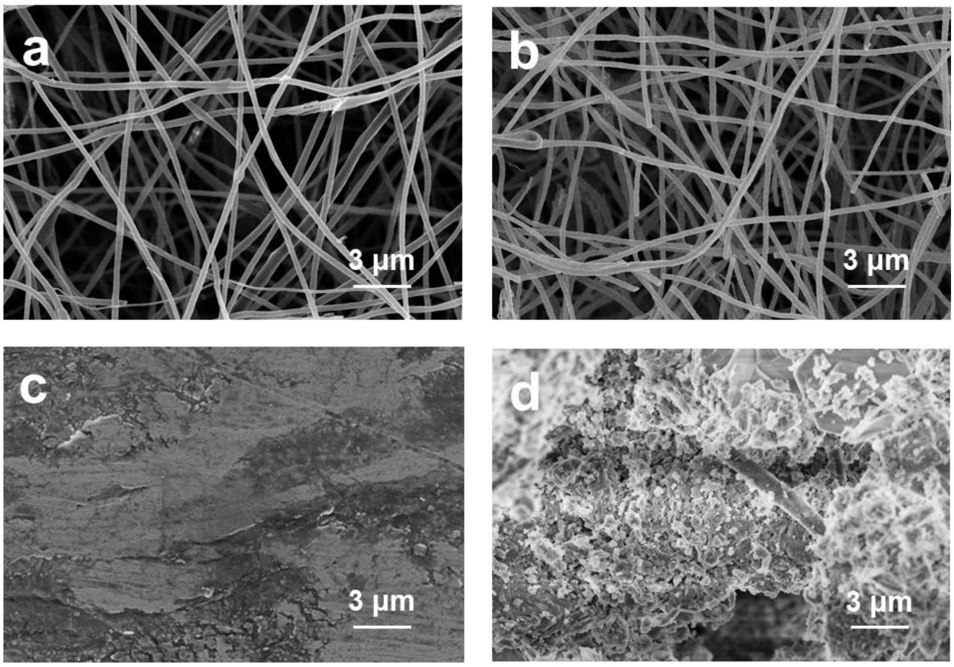

Fig. S14 SEM images of (**a**) initial N-OPCNF cathode. (**b**) N-OPCNF cathode at 40 A g^{-1} after 200,000 cycles. (**c**) initial Zn anode. (**d**) Zn anode at 40 A g^{-1} after 200,000 cycles

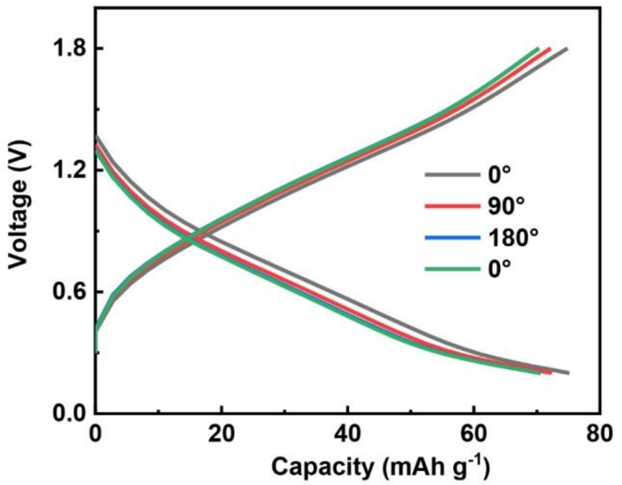

Fig. S15 GCD profiles of N-OPCNF pouch cell under different bending angles

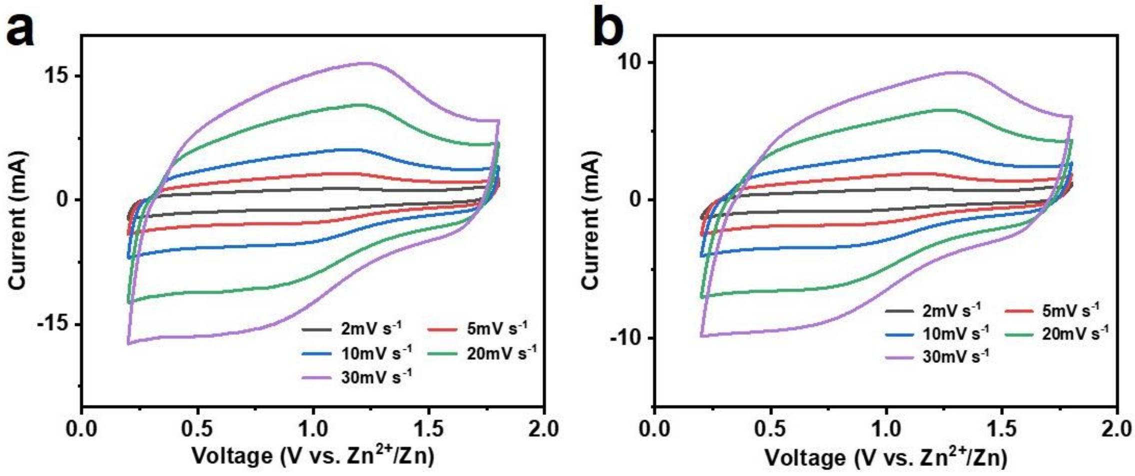

Fig. S16 CV curves at various scan rates for (**a**) N-OPCNF and (**b**) OPCNF

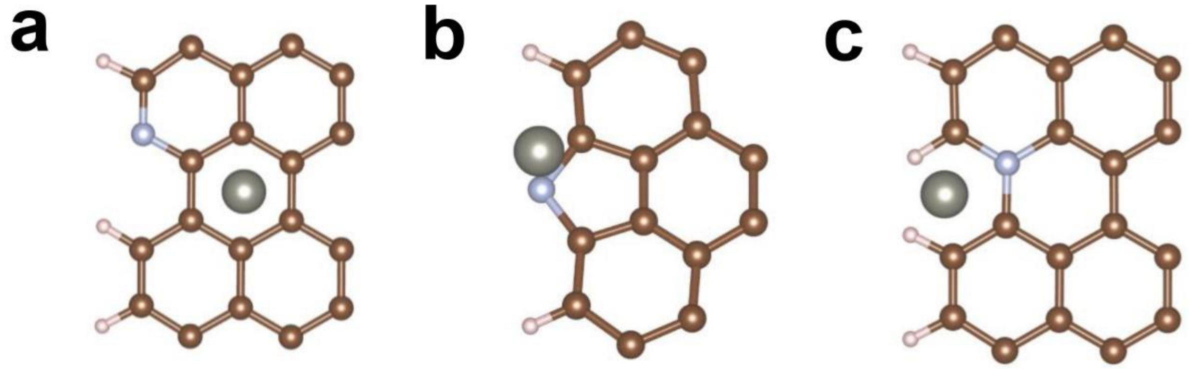

Fig. S17 Optimized structures of Zn adsorption on the surfaces of (**a**) N6-doped graphene, (**b**) N5-doped graphene, and (**c**) NQ-doped graphene

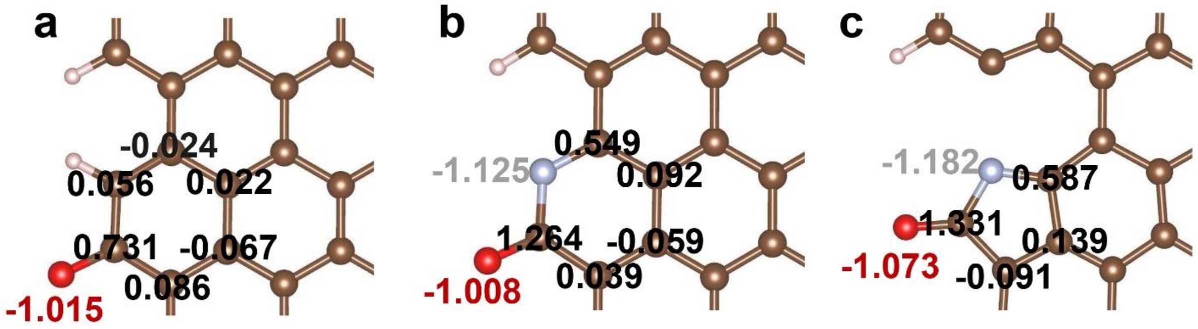

Fig. S18 Charge distributions for graphene in its optimized structures. (**a**) carbonyl functionalized graphene, (**b**) adjacent N6-doped and carbonyl functionalized graphene and (**c**) adjacent N5-doped and carbonyl functionalized graphene. Positive and negative value marks respectively show lost and gained electron number. Brown, white, and red balls represent C, H, and O atoms, respectively

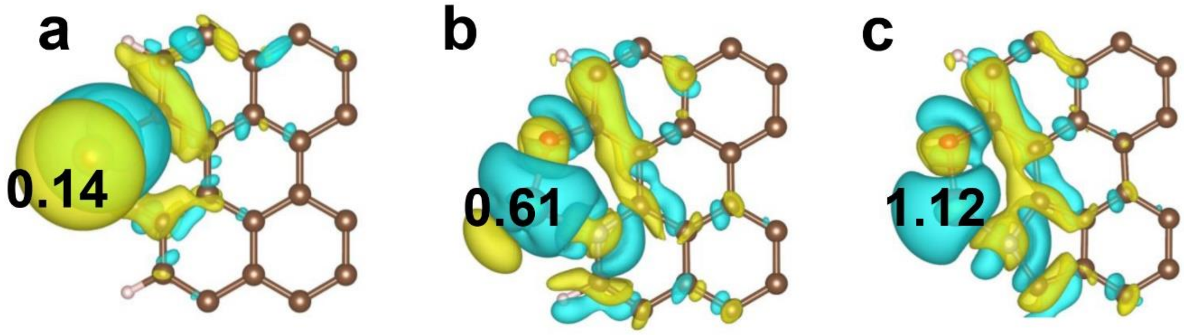

Fig. S19 Charge density difference of Zn adsorption on the (**a**) carbonyl functionalized graphene, (**b**) alternate site N6-doped and carbonyl functionalized graphene, (**c**) alternate site N5-doped and carbonyl functionalized graphene. yellow and cyan contour indicates augmented and reduced charge, respectively. Positive value mark shows lost electron number

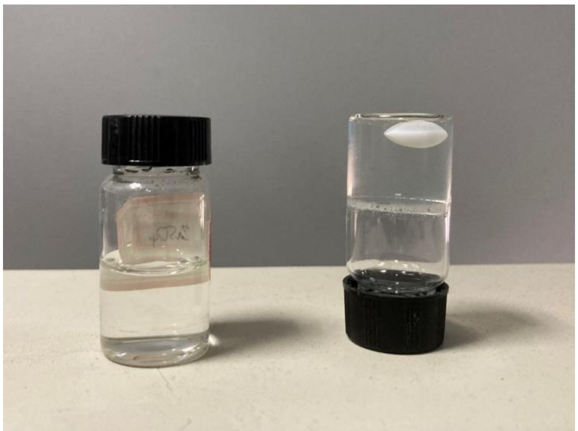

Fig. S20 Optical images of 1 M $ZnSO_4$ aqueous solution (left) and gelatin/$ZnSO_4$ gel electrolyte (right)

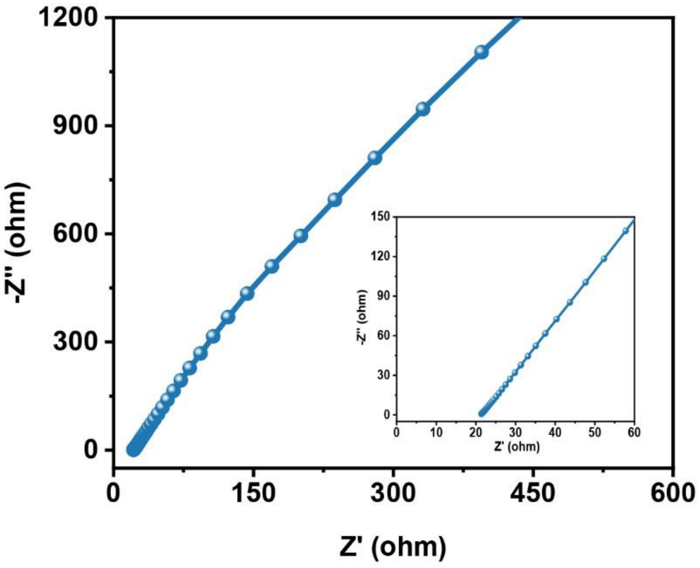

Fig. S21 AC impedance spectra of the gelatin/$ZnSO_4$ gel electrolyte

Calculation method of ionic conductivity of the gelatin/$ZnSO_4$ gel electrolyte

The ionic conductivity (σ) was calculated as a function of the ohmic resistance (R), thickness (L), and area (A) of the gel electrolyte according to the equation:

$$\sigma = \frac{L}{AR}$$

Table S4 Electrochemical performances of reported carbon-based cathode materials applied for ZIHCs

**Materials**	**Synthetic method**	**Rate capacity (mAh/g) / current density (A/g)**	**Capacity retention**	**Refs.**
HCS	Carbonization polymers	86.8/0.5; 65.1/1; 53.3/2; 49.4/3; 47.1/4	98 % at 1 A/g after 15000 cycles	[S1]
PSC-A600	KOH activation	140/1; 115/5; 95/10	92.2 % at 10 A/g after 10000 cycles	[S2]
LDC	Intercalator-guided pyrolysis	101/1; 65/5; 51/10; 42.8/20	81.3 % at 5 A/g after 6500 cycles	[S3]
MCHSs	Template method	121/1; 105/5; 96.9/10	96 % at 1 A/g after 10000 cycles	[S4]
AC	Commercial material	121/0.1; 85/1; 58/5; 41/20	91 % at 1 A/g after 10000 cycles	[S5]
P&B-AC	One-pot doping calcination	169.4/0.5; 130/2; 103/5; 84/10	88 % at 10 A/g after 30000 cycles	[S6]
N-HPC	Activation	136.8/0.1; 110.9/0.5; 102.6/1; 76.1/5; 66.5/10	90.9 % at 1 A/g after 5000 cycles	[S7]
OPC	Calcination	132.7/0.2; 99.1/0.5; 79/1; 66/2; 61/3; 54.5/4	87.6 % at 1 A/g after 10000 cycles	[S8]
N-OPCNF	Electrospinning	136/0.1; 101/0.5; 93/1; 76/10; 63/30; 57/50	99.2 % at 40 A/g after 200000 cycles	**This work**

Supplementary References

[S1] S. Chen, L. Ma, K. Zhang, M. Kamruzzaman, C. Zhi et al., A flexible solid-state zinc ion hybrid supercapacitor based on co-polymer derived hollow carbon spheres. J. Mater.

Chem. A **7**(13), 7784-7790 (2019). https://doi.org/10.1039/C9TA00733D

[S2] Z. Li, D. Chen, Y. An, C. Chen, L. Wu et al., Flexible and anti-freezing quasi-solid-state zinc ion hybrid supercapacitors based on pencil shavings derived porous carbon. Energy Storage Mater. **28**, 307-314 (2020). https://doi.org/10.1016/j.ensm.2020.01.028

[S3] Y. Lu, Z. Li, Z. Bai, H. Mi, C. Ji et al., High energy-power Zn-ion hybrid supercapacitors enabled by layered B/N co-doped carbon cathode. Nano Energy **66**, 104132 (2019). https://doi.org/10.1016/j.nanoen.2019.104132

[S4] P. Liu, W. Liu, Y. Huang, P. Li, J. Yan et al., Mesoporous hollow carbon spheres boosted, integrated high performance aqueous Zn-ion energy storage. Energy Storage Mater. **25**, 858-865 (2020). https://doi.org/10.1016/j.ensm.2019.09.004

[S5] L. Dong, X. Ma, Y. Li, L. Zhao, W. Liu et al., Extremely safe, high-rate and ultralong-life zinc-ion hybrid supercapacitors. Energy Storage Mater. **13**, 96-102 (2018). https://doi.org/10.1016/j.ensm.2018.01.003

[S6] Y.G. Lee, G.H. An, Synergistic effects of phosphorus and boron co-incorporated activated carbon for ultrafast zinc-ion hybrid supercapacitors. ACS Appl. Mater. Interfaces **12**(37), 41342-41349 (2020). https://doi.org/10.1021/acsami.0c10512

[S7] P. Liu, Y. Gao, Y. Tan, W. Liu, Y. Huang et al., Rational design of nitrogen doped hierarchical porous carbon for optimized zinc-ion hybrid supercapacitors. Nano Res. **12**(11), 2835-2841 (2019). https://doi.org/10.1007/s12274-019-2521-6

[S8] Y. Zheng, W. Zhao, D. Jia, Y. Liu, L. Cui et al., Porous carbon prepared via combustion and acid treatment as flexible zinc-ion capacitor electrode material. Chem. Eng. J. **387**, 124161 (2020). https://doi.org/10.1016/j.cej.2020.124161
